# Clinical utility of inpatient monitoring for reduced fetal movements in the absence of fetal pathology

**DOI:** 10.1007/s00404-026-08579-w

**Published:** 2026-09-24

**Authors:** Gilbert Georg Klamminger, Felix Hässlin, Kathrin Stewen, Bernadette Eser, Annette Hasenburg, Yaman Degirmenci, Joscha Steetskamp

**Affiliations:** Department of Obstetrics and Gynecology, University Medical Center of the Johannes Gutenberg University Mainz, Langenbeckstraße, 1, 55131 Mainz, Germany

**Keywords:** Reduced fetal movements, Fetal assessment, Inpatient monitoring

## Abstract

**Background:**

Evidence on the follow-up of women with reduced fetal movements (RFM) and physiological fetal parameters at initial assessment is limited. We evaluated whether inpatient monitoring provides additional clinical value in this population.

**Material and methods:**

In this retrospective study, women with a first episode of RFM and physiological fetal assessment (by means of cardiotocography, single deepest vertical pocket, estimated fetal weight, and umbilical artery Doppler) between January 2017 and December 2025 were identified and their clinical course and inpatient monitoring was analyzed. Patients with fetal pathology, recurrent RFM, or multiple gestation were excluded. Clinical outcomes were compared with a matched control cohort.

**Results:**

Of 154 identified cases with RFM, 78 women met our inclusion criteria. Median gestational age was 36 + 2 weeks (IQR 33 + 0–37 + 5) and median hospital stay 1.5 days (IQR 1–2). In 74 (94.87%) patients, inpatient monitoring yielded no additional findings and did not change management; in 4 (5.13%) cases, changes in the clinical course occurred, but were attributable to independent obstetric factors (hypertension, premature rupture of membranes, impending uterine rupture, recurrent RFM). No distinct differences in birth outcomes between patients with RFM and controls were observed.

**Conclusion:**

In women presenting with RFM and no evidence of fetal compromise, inpatient monitoring rarely influenced clinical management. Outpatient follow-up with counselling may be a feasible alternative pending further validation.

## What does this study adds to the clinical work

**Table:** 

In women presenting with reduced fetal movements, inpatient monitoring rarely influenced clinical management, suggesting that outpatient follow-up may be a feasible approach for carefully selected low-risk patients, pending further validation.	

## Introduction

Up to 40% of pregnancies report perception of reduced fetal movements (RFM) - it is a common reason for presentation in acute maternity assessment settings accounting for roughly 6.1%–22.6% of the caseload [1–3]. RFM are defined by maternal perceptions of reduced strength, frequency, or alterations in diurnal patterns in fetal movements rather than by objective counting-based measures [4] - as stated in the German guideline *Fetal Assessment in Pregnancy (Indication and Methodology for Fetal Monitoring in a Low-risk Population)* a routine counting of fetal movements is not recommended [5]. Relative to women who did not report RFM, patients with an episode of RFM are more likely to be younger, nulliparous, have an anterior placenta, and exhibit a higher body mass index; they demonstrate an increased likelihood of undergoing labor induction, also attributable to the presence of RFM itself [6–8]. Women with RFM demonstrate substantially higher odds of stillbirth (OR 3.44, 95% CI 2.02–5.88) alongside an increased likelihood of delivering small-for-gestational-age (SGA) infants [9].

There is a lack of distinct evidence-based recommendations in Germany addressing the management of women presenting with RFM [10]. Internationally, maternal assessment of patients presenting with RFM is defined in the Royal College of Obstetricians and Gynaecologists (RCOG) guideline *Reduced Fetal Movements* and in Australian guidelines (developed by institutions or local healthcare districts), such as the guideline from the Perinatal Society of Australia and New Zealand. Adopting a-risk stratified approach, incorporating gestational age and coexisting risk factors for stillbirth (e.g., fetal growth restriction or placental insufficiency), these recommendations advocate the utilization of CTG (cardiotocography) alongside ultrasonographic evaluation (fetal biometry, amniotic fluid index (AFI), and umbilical artery Doppler) in women at more than 28+0 weeks’ gestation presenting with RFM [11, 12]. Clinical assessment of RFM at hospital presentation typically often includes blood pressure measurement, symphysis–fundal height, CTG, amniotic fluid volume assessment, and detailed ultrasound (fetal biometry and umbilical artery Doppler). Current routine management pathways may also involve hospital admission, induction of labor, and prelabor caesarean delivery [6, 13]. However, substantial variability in clinical practice across healthcare providers and maternity units persists [4, 10, 11, 14], and a significant proportion of women may not receive care that is consistent with evidence-based guidelines. Against this paucity of robust evidence, clinicians must balance minimizing unnecessary tests and interventions with the timely identification of fetuses at risk of stillbirth or adverse perinatal outcomes [7, 10].

As highlighted in the current RCOG guidelines, there is a distinct lack of studies examining the follow-up of women with normal findings [12]. Furthermore, a Cochrane systematic review by Hofmeyer et al. identified the distinction between women with antecedent risk factors and those without such predispositions as a key consideration [10]. In Germany, several hospitals traditionally recommend and implement inpatient admission for 24 h of fetal monitoring, including repeated CTG surveillance, followed by discharge once regular fetal movements resume. However, the rationale for hospital admission in women presenting solely with RFM in the absence of abnormal CTG or ultrasound findings is largely based on clinical experience rather than robust scientific evidence [14, 15].

In this study, we conducted a retrospective analysis to determine whether (a) inpatient monitoring, including repeated CTG surveillance in patients presenting with RFM and physiological initial fetal assessment (CTG, single deepest vertical pocket (SDP), estimated fetal weight, umbilical artery Doppler) provided additional diagnostic information or necessitated further clinical intervention, and (b) to compare maternal and neonatal delivery outcomes of patients with RFM and a physiological initial fetal assessment with a regular control cohort.

## Material and methods

To identify and evaluate patients presenting with RFM who underwent inpatient monitoring, the internal clinical database of our university-based tertiary referral maternity hospital (approximately 2000 deliveries per year) was searched for cases coded under ICD-10-GM O36.8 (“Maternal care for other specified fetal problems”) who had an inpatient stay of at least one night between January 2017 and December 2025. Retrieved records were screened manually, and eligible cases (criteria: presentation with RFM) were included on a case-by-case basis following detailed review of the medical charts (GGK, FH). In accordance with local clinical practice, RFM was defined as a maternal subjective perception of decreased or absent fetal activity prompting clinical evaluation. A recurrent episode of RFM was defined as a subsequent presentation occurring more than 24 h after an initial episode, with normal perception of fetal movements in the intervening period. At antenatal presentation, women with RFM were routinely assessed by board-certified obstetricians or experienced residents (obstetrics and gynecology) with expertise in fetal ultrasonography and CTG interpretation. Clinical evaluation included a comprehensive medical and obstetrical history, urine analysis for proteinuria, blood pressure measurement with assessment for hypertension (> 140/90 mmHg), CTG, and ultrasound examination comprising fetal biometry (normal range: between 10% and 95%), SDP (normal range 2–8 cm), and umbilical artery Doppler assessment (normal pulsatility index [PI] < 95th percentile). In cases where the patient had attended the clinic within the preceding 14 days, previously recorded fetal biometry values were used, and only CTG, SDP, and Doppler assessment were repeated. The maternity unit operates a 24-h service, staffed also by midwives and a senior physician on call.

The primary outcome of this retrospective hypothesis-generating study was defined as the proportion of cases in which repeated CTG surveillance during inpatient monitoring/hospitalization provided additional diagnostic information or resulted in a change in clinical management among women presenting with reduced fetal movements (RFM), who had an initially normal fetal assessment. Inclusion criteria comprised presentation with a first episode of RFM, a physiological initial fetal assessment (CTG, SDP, estimated fetal weight, umbilical artery Doppler) and a gestational age between 24 + 0 and 39 + 0 weeks. Intercurrent transient infectious conditions (e.g., urinary tract infection, influenza-like illness, or diarrhoea) or history of outpatient suspicious CTG patterns were not considered as exclusion criteria. Exclusion criteria included evidence of fetal pathology at initial assessment (abnormal CTG, abnormal umbilical artery Doppler, abnormal SDP, or abnormal estimated fetal weight), multiple gestation, recurrent presentations with RFM, premature rupture of membranes/onset of labor prior to clinical visit, gestational age >39+0 weeks (as potential induction is here more routinely performed in clinical routine also due to concurrent, independent risk factors) and insufficient data (> 30% lacking). Pharmacotherapy during pregnancy was not recorded, since the main aim of the study was not to examine a potential relationship between medication use and RFM. Finally, data on delivery outcomes (e.g., mode of delivery) as well as postpartum outcomes (e.g., birth weight) were collected by searching the clinical information system.

To evaluate whether inpatient monitoring yields additional diagnostic information or requires further clinical intervention, we defined two distinct cohorts: *Cohort A* included patients without maternal risk factors while *cohort B* also included patients with one or more of the following maternal risk factors: BMI > 30, age > 40 years, preeclampsia, hypertonus, SGA, FGR, (gestational) diabetes, intrahepatic cholestasis of pregnancy, previous intrauterine fetal death (IUFD). Delivery outcomes of women with RFM were compared to a contemporaneous control cohort of women who delivered during the same time period. Controls were retrospectively drawn at random from a comprehensive delivery database covering the study period. A systematic sampling approach was used, selecting every 5th entry, which comprised both low- and high-risk pregnancies [6].

### Statistical analysis

Descriptive statistics and statistical analyses were carried out using GraphPad (Boston, Version 11.0.0, MA 02110, USA). Parameters with < 30% missing values within a given group were considered acceptable and were included in the analysis without further adjustment. After an initial normality check (Shapiro–Wilk test), variables were assessed using either the unpaired t-test (from Gaussian Population), or the Mann–Whitney test; categorical variables were analyzed employing the Fisher’s exact test. A *p*-value of < 0.05 was considered the threshold for statistical significance.

## Results

Using the predefined search strategy, 263 cases were initially identified. Of these, 154 women presented with RFM. Other cases identified through the search that involved solely history of trauma during pregnancy, infectious diseases, or suspicious CTG performed elsewhere were excluded from further consideration.

**Fig. 1:**
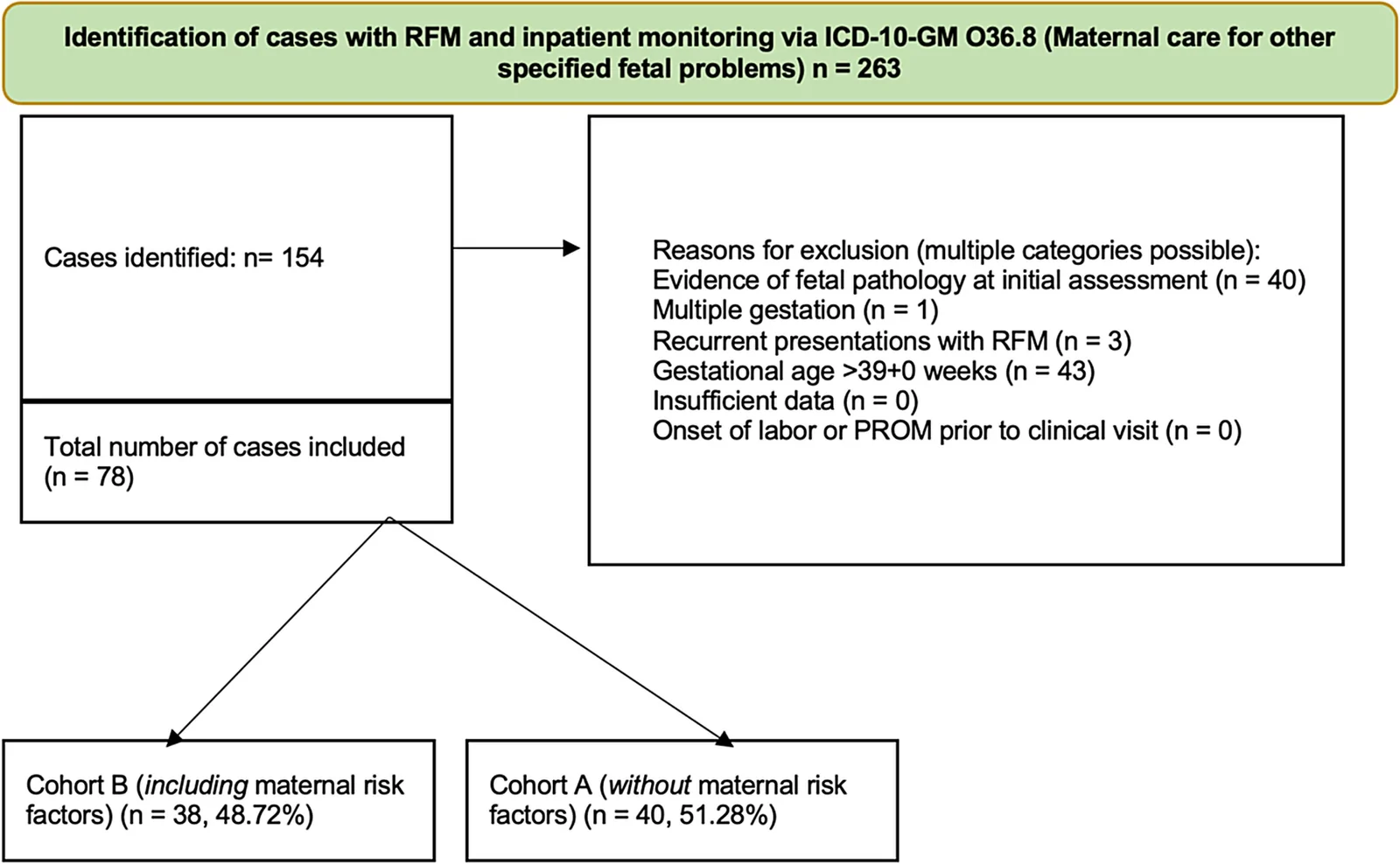
Study flow diagram of patient selection

Among the 154 RFM cases, 78 patients met the strict inclusion criteria for analysis. Exclusions comprised (multiple criteria per patient possible): 40 patients with evidence of fetal pathology at initial assessment, one patient with multiple gestation, three patients with recurrent RFM, and 43 patients with a gestational age > 39 + 0 weeks. Of the 78 included patients, 40 (51.28%) were assigned to cohort A and 38 (48.72%) to cohort B (see Fig. 1).

In our highly selected cohort of 78 patients without evidence of fetal pathology at admission, the median gestational age at initial presentation was 36w + 2 (IQR: 33w + 0 to 37w + 5), the duration of hospitalization was 1.5 days (IQR: 1–2). Patients had a median age of 32 (29–35) years and an average BMI of median 29.19 (IQR: 23.72–33.34); most women had an anterior placenta localization 39 (50%). Across 74 patients (94.87%), repeated CTG evaluations during hospitalization did not reveal new findings and did not prompt any modification of treatment. Four (5.13%) patients experienced changes in their clinical status, necessitating individualized clinical management; three (3.85%) of these patients belonged to cohort B (with maternal risk factors). One (1.28%) patient (39 years, G2P1, 38 + 3 weeks) developed premature rupture of membranes (PPROM) on the following day after admission and subsequently underwent delivery. In another patient (41 years, G1, 38 + 2 weeks), labor was induced due to poorly controlled pre-existing hypertension, which had already been evident at admission with blood pressure readings of approximately 150/100 mmHg. A third patient (35 years, G5P4, 35 + 4 weeks) required delivery due to suspected impending uterine rupture in the context of a prior cesarean section.

The remaining case (21 years, G1, 38 + 4 weeks) - the only patient from cohort A - had a prolonged hospital stay of four days due to recurrent RFM and was subsequently transferred to another hospital for further observation owing to internal capacity constraints.

Among individual clinical characteristics (age, parity, BMI), only BMI differed significantly in the RFM group (higher BMI of median 29.19 kg/m^2^; *p* = 0.0006, Mann–Whitney test) compared with the a priori defined control group (Table 1). Regarding birth outcomes: Although the 5-min Apgar scores differed between the groups (*p* = 0.0371, Mann–Whitney test), the direction of this difference did not indicate poorer neonatal condition in the RFM group (median 5-min Apgar RFM group: 10 (IQR: 9–10) versus median 5-min Apgar control group: 10 (IQR: 9–10)). Other parameters evaluated, including preterm delivery, mode of birth, birth weight, umbilical artery pH (UA pH), 10-min Apgar score - did not differ significantly between groups (Table 1). Birth data were available for 59 patients in the RFM cohort.

**Table 1:** Clinical characteristics and perinatal outcomes: RFM versus control group

Variables	RFM group (*n* = 78)	Control group (*n* = 95)	*p*- value
Gestational age at initial presentation (weeks + days; median, IQR)	36w+2 (33w+0—37w+5)	−	−
Placental location as assessed by ultrasound			−
- anterior	39 (50%)	−	
- posterior	35 (44.87%)	−	
- lateral	3 (3.85%)	−	
- fundal	1 (1.28%)	−	
Maternal age (years), mean ± SD	31.8 (5.8)	31.5 (5.1)	0.7423^1^
Body mass index (BMI, kg/m^2^), median (IQR)	29.19 (23.72–33.34)	23.34 (20.74–26.98)	0.0006^2^
Parity			0.2174^2^
- 0	52 (66.67%)	50 (53.76%)	
- 1	13 (16.67%)	32 (34.41%)	
- 2	11 (14.10%)	6 (6.45%)	
- 3	0	3 (3.23%)	
- 4	1 (1.28%)	2 (2.15%)	
- 5	0	0	
- 6	1 (1.28%)	0	
Preterm delivery (< 37 + 0 weeks), n (%)	3 (5.09%)	10 (10.53%)	0.3722^3^
Mode of delivery, n (%)			0.5956^3^
- Spontaneous vaginal delivery	34 (57.63%)	53 (55.79%)	
- Unplanned caesarean section	15 (25.42%)	19 (20%)	
- Planned caesarean section	7 (11.86%)	16 (16.84%)	
- Vacuum-assisted delivery	3 (5.08%)	7 (7.37%)	
Umbilical artery pH, median (IQR)	7.280 (7.220–7.330)	7.280 (7.210–7.340)	0.9692^2^
Birth weight (g), median (IQR)	3320 (3000–3620)	3360 (2950–3680)	0.8888^2^
Apgar score at 5 min, median (IQR)	10 (9–10)	10 (9–10)	0.0371^2^
Apgar score at 10 min, median (IQR)	10 (10–10)	10 (10–10)	0.4486^2^

^1^Unpaired t-test, ^2^Mann-Whitney test, ^3^Fisher’s exact test. *SD* Standard deviation, *IQR* Interquartile Range, Parameters with < 30% missing values within a given group were considered acceptable. Birth data were available for 59 patients in the RFM cohort

## Discussion

In this retrospective analysis, we evaluated the clinical course of a highly selected cohort of 78 patients presenting with RFM without evidence of fetal compromise on initial assessment by means of CTG, biometry, Doppler, and SDP - a population of particular clinical relevance, as optimal surveillance strategies remain undefined [12].

For most patients (94.87%), inpatient monitoring with repeated CTG assessment did not provide additional prognostic value and did not alter clinical management. Among the four patients (5.13%) with a change in clinical course, these changes were attributable either to the physiological progression of pregnancy (one case of PPROM) or to pre-existing maternal risk factors (hypertension and suspected impending uterine rupture at presentation), which independently warranted clinical intervention irrespective of RFM. One (1.28%) patient reported recurrent RFM during hospitalization; unfortunately, follow-up data on this case were unavailable. Notably, recurrent RFM is a recognized risk factor for adverse outcomes and was predefined as an exclusion criterion in this study [16, 17]. In a hypothetical alternative pathway, this patient could have been adequately educated on normal fetal movement patterns/multiple episodes of RFM and discharged after initial presentation - she would then subsequently have re-presented with recurrent RFM, at which point escalation from low-risk to high-risk care with intensified monitoring would have been indicated. This scenario is of distinct clinical importance, as it demonstrates that the maternal risk status is dynamic over the course of pregnancy. This hypothesis supports an alternative, yet feasible management approach based on patient education and re-presentation if needed, rather than routine inpatient monitoring for all patients undergoing a first episode of RFM. Current RCOG guidelines explicitly emphasize education on physiological fetal movement patterns and maternal awareness of individual activity: Women should be counseled that fetal movements are best assessed subjectively, do not decrease in the third trimester, are not influenced by maternal BMI or fetal presentation, and should be reported regardless of placental position [12, 18–21].

In our cohort, BMI was higher in the RFM group, consistent with a systematic review by Bradford et al., which found an association between maternal body size and increased presentation with RFM [20]. Overall, birth outcomes among patients with RFM did not indicate a distinctly worse neonatal outcome compared with the control group, also in line with existing literature indicating that most pregnancies complicated by RFM nevertheless result in favorable neonatal outcomes [12].

In the United Kingdom, the “Saving Babies’ Lives” care bundle promotes measures (among others: awareness of RFM, reducing smoking, management of pre-existing diabetes) as an initiative to reduce stillbirth rates [2, 7, 22]. While stillbirth rates have declined in countries, such as the UK and Norway [4], this trend has not been observed in Germany, where stable or increasing rates may partly reflect changing maternal characteristics, including advanced maternal age and higher prevalence of obesity, as well as methodological and legal aspects (e.g., the documentation of fetal deaths, irrespective of birth weight, occurring at or beyond 23 completed weeks of gestation since 2018) [23–26]. Evidence-based strategies to reduce stillbirth are highly desirable and may support the development of nationwide guidelines to standardize care and improve access to evidence-based management. Nevertheless, it is essential to avoid unnecessary interventions and iatrogenic induction of increased maternal anxiety [12, 27]. In this context, the widely recognized AFFIRM trial did not demonstrate a reduction in stillbirth rates following implementation of a structured so-called “RFM care package,” which included delivery for women > 37 weeks’ gestation in the presence of estimated fetal weight or abdominal circumference below the 10th centile, AFI <2 cm, abnormal CTG, or recurrent RFM [28]. It is within this area of tension that our exploratory analysis is situated, providing a scientific basis for a larger and prospective study evaluating optimal surveillance strategies (e.g., inpatient monitoring versus outpatient management) in patients with RFM and lack of fetal pathology at initial assessment - all with the ultimate aim of maximizing patient safety while minimizing unnecessary anxiety and interventions.

Several limitations of this retrospective study should be acknowledged. (a) The cohort size was relatively small, as of 154 patients presenting with RFM, 76 were excluded due to evidence of fetal pathology at initial assessment. However, this proportion is consistent with previous studies, in which a substantial percentage of patients (ranging from 15.2% to 43%) even undergo prelabor cesarean delivery or induction due to abnormal findings (e.g., CTG or amniotic fluid index) at first presentation [2, 6]. That said, the primary objective of this retrospective, hypothesis-generating study was to evaluate the clinical utility of inpatient monitoring rather than to assess perinatal outcomes. For the latter purpose, inclusion of an additional control group - specifically, women presenting with RFM and reassuring initial assessments who had an outpatient management - would be necessary to allow a formal evaluation of non-inferiority between management strategies. Such an analysis, however, is beyond the scope and intent of the present study. (b) This study was designed to generate preliminary findings and hypotheses for future prospective research rather than to test a prespecified effect size or to provide definitive statistical inference. Accordingly, the available sample was determined by the extent of accessible clinical data, which was limited to routine hospital records, rather than a priori sample size calculation. (c) Even with the implementation of stringent exclusion criteria, the possibility of residual confounding cannot be fully excluded. (d) Given the known association between RFM and placental pathology, histopathological evaluation of the placenta would be of particular interest in a cohort without clinical fetal pathology. However, placental histology was available for only one patient in this study; this aspect warrants further investigation in future studies.

## Conclusion

Taken together, our findings suggest that in women presenting with RFM and absent fetal distress, inpatient monitoring with repeated CTG rarely resulted in changes in clinical management. Clinical worsening appeared to be uncommon and, when present, was largely associated with independent factors. These observations indicate that outpatient management with appropriate patient education may be a feasible approach in selected cases; however, this should be interpreted with caution and requires confirmation in prospective studies.

## Data Availability

The datasets generated and/or analyzed during the current study are restricted due to ethical restrictions.
